# Association of 10-Year C-Reactive Protein Trajectories With Markers of Healthy Aging: Findings From the English Longitudinal Study of Aging

**DOI:** 10.1093/gerona/gly028

**Published:** 2018-02-15

**Authors:** Camille Lassale, G David Batty, Andrew Steptoe, Dorina Cadar, Tasnime N Akbaraly, Mika Kivimäki, Paola Zaninotto

**Affiliations:** 1 Department of Epidemiology and Public Health, London, UK; 2 Department of Behavioural Science and Health, University College London, London, UK; 3 MMDN, University Montpellier, EPHE, INSERM, Montpellier, France

**Keywords:** Inflammation, Successful aging, Cardiovascular, Physical function, Mental health

## Abstract

**Background:**

Elevated systematic inflammation is a hallmark of aging, but the association of long-term inflammation trajectories with subsequent aging phenotypes has been little examined. We assessed inflammatory marker C-reactive protein (CRP) repeatedly over time and examined whether long-term changes predicted aging outcomes.

**Methods:**

A total of 2,437 men and women aged 47–87 years at baseline (1998–2001) who were participants in the English Longitudinal Study of Ageing had CRP measured on two or three occasions between 1998 and 2009. Inflammation trajectories were computed using latent-class growth mixture modeling and were related to aging outcomes measured in 2012/2013: physical functioning, cardiometabolic, respiratory, mental health, and a composite “healthy aging” outcome.

**Results:**

Four CRP trajectories were identified as follows: “stable-low” (71 per cent of the sample) with baseline mean 1.33 mg/L remaining <3 mg/L; “medium-to-high” (14 per cent) with baseline 2.7 mg/L rising to 5.3 mg/L; “high-to-medium” (10 per cent) with baseline 6.6 mg/L decreasing to 2.4 mg/L; and “stable-high” (5 per cent) with levels from 5.7 to 7.5 mg/L. Relative to the stable-low trajectory, individuals in the medium-to-high had a higher risk of limitations in basic activities of daily living (ADL, odds ratio; 95% confidence interval: 2.09; 1.51, 2.88), instrumental ADL (1.62; 1.15, 2.30), impaired balance (1.59; 1.20, 2.11) and walking speed (1.61; 1.15, 2.24), arthritis (1.55; 1.16, 2.06), hypertension (1.57; 1.21, 2.04), obesity (1.95; 1.36, 2.80), poor respiratory function (1.84; 1.36, 2.50), and depression (1.55; 1.13, 2.12). A lower odds of healthy aging was observed in people in the medium-to-high (0.57; 0.40, 0.79) and stable-high (0.50; 0.27, 0.91) trajectories.

**Conclusions:**

Older people who displayed an elevation in CRP levels over a decade experienced an increased risk of adverse aging outcomes.

Biological aging is characterized by a chronically active immune system (1, 2). Elevated levels of inflammatory markers in older age, in the absence of acute infection, have been described in many studies with the term of “inflammaging” coined a decade ago (3). Proposed mechanisms leading to a state of chronic systemic inflammation include increased adiposity, oxidative stress, glycation, immunosenescence, epigenetic and hormonal dysregulation, proinflammatory lipid signaling, mitochondrial and telomere dysfunction, and long-term infections (4, 5).

There is growing evidence from longitudinal observational studies of associations between elevated levels of inflammatory cytokines such as interleukin-6 (IL-6), tumor necrosis factor-α (TNF-α), or the acute phase C-reactive protein (CRP), and a range of adverse aging outcomes including accelerated vascular aging, atherosclerosis, bone and muscle loss, and cognitive impairment (6). Among the range of available biomarkers of inflammation, CRP is easily assessed by standardized laboratory assays, the most widely available in clinical practice and the most extensively studied inflammatory marker (7). Inflammatory indices, like many biomarkers of risk, are time-varying, yet, in most studies linking their measurement with aging outcomes, they have been assessed at a single point in time (8). Repeat measurements of inflammatory biomarkers over a long period of time are crucial to better understand the relationship between long-term inflammation and later onset of impairment in physical, cardiometabolic, respiratory, or mental health.

Benefiting of a long period of follow-up in a population-based cohort study with repeat measures of CRP available across the older age life course, the aim of the present analyses was to identify 10 year trajectories of CRP levels in English older adults and assess the association of these trajectories with a range of age-related phenotypes.

## Methods

The English Longitudinal Study of Ageing (ELSA) is an on-going, nationally representative prospective cohort study of men and women living in England who were aged ≥50 years at recruitment in 2002/2003 (wave 1) (9). This original sample was drawn from the Health Surveys for England (HSE), a collection of population-based cross-sectional studies conducted in 1998, 1999, and 2001 (“wave 0”—referred to hereafter as our baseline). Because we used wave 0 measurements, participants in the present analysis had a minimal age of 47 years. Data are collected at waves every 2 years in ELSA using computer-assisted personal interviews (CAPI) and self-completion questionnaires, with additional nurse visits every other wave (ie every 4 years) for the assessment of biomedical data. CRP measurements were made during the nurse visits from wave 0 (1998–2001), wave 2 (2004–2005), and wave 4 (2008–2009) with aging outcomes assessed at wave 6 (2012–2013). Ethical approval for all data collection was granted from NHS Research Ethics Committees under the National Research and Ethics Service and all participants provided informed consent (10).

### Measurement of CRP and Covariates

At each nurse visit, a blood sample was drawn. High sensitivity serum CRP (mg/L) was analyzed using the N Latex CRP mono Immunoassay on the Behring Nephelometer II Analyzer (Dade Behring, Milton Keynes, UK) (11). For the purposes of the present analyses, CRP was treated as a continuous variable. We excluded study members with values of CRP > 10 mg/L, reasoning that they may be cases of acute inflammation and immune activation due to current infection rather than chronic inflammation.

We used covariates collected at baseline: age, sex, educational level [low (no qualification), moderate (up to high school diploma), and high (university degree or higher)], current smoking status (yes/no), and total physical activity (four levels from low to high). Use of anti-inflammatory and antihypertensive medication was self-reported. These constitute the set of common covariates to all models. We used additional covariates measured at wave 1, specific to each outcome, described hereafter.

### Measurement of Physical Function and Limitation in Activities of Daily Living

Gait speed (m/second) was measured directly with respondents aged 60 years and older walking a distance of 8 feet (2.4 m) twice: the mean speed (m/second) of the two trials was used. Static balance was evaluated in three separate and progressively more difficult tests: feet side-by-side stand for 10 seconds; preferred heel and toe side-by-side stand (semitandem) for 10 seconds; and preferred heel in front of toe stand (full tandem) for 10 seconds if aged ≥70 years (30 seconds if aged <70 years). Failure was denoted by an inability to complete one or more of these balance tests. Grip strength (kg) was measured by asking the respondents to maximally squeeze a hand-held dynamometer for 2 seconds on three occasions per hand; the value used in analyses was the average of the three measurements from the dominant hand. Lower body strength was based on the capacity to rise from a chair with arms across the chest to a full standing position on five occasions for persons aged ≥70 years (ten occasions for persons <70 years). Impairment was defined as failing to complete 5 (those aged ≥70) or 10 rises (aged <70) or not attempted as did not feel safe. Disability was defined as reporting any limitations out of six activities of daily living (ADL) such as dressing, including putting on shoes and socks; walking across a room; bathing or showering; eating, such as cutting up food; getting in or out of bed; using the toilet, including getting up and down; and any of the seven instrumental ADL (IADL) such as using a map to ascertain how to get around in a strange place; preparing a hot meal; shopping for groceries; making telephone calls; taking medications; doing work around the house or garden; and managing money, such as paying bills and keeping track of expenses. Doctor-diagnosed arthritis was self-reported. “Baseline” report of arthritis, limitation with ADL, and IADL were collected in a similar way at wave 1 and used as covariates to account for the presence of dysfunction at baseline.

### Measurement of Cardiometabolic Health and Respiratory Function

Body weight was measured using TANITA electronic scales (Tokyo, Japan) without shoes and in light clothing; height was measured using a stadiometer with the Frankfort plane in the horizontal position (Seca Leicester stadiometer, Birmingham, UK). Body mass index (BMI) was calculated as weight (kg)/height (m) squared and obesity defined as a BMI ≥ 30 kg/m^2^ (12). Systolic and diastolic blood pressure (SBP and DBP) was measured with an Omron HEM-907 BP monitor three times in the sitting position after 5 minute rest before the first measurement and leaving 1 minute between each reading. An average of the second and third BP recordings was used for the present analyses. Hypertension was defined as SBP ≥ 140 mm Hg and/or DBP ≥ 90 mm Hg, and/or use of antihypertensive medication. High density lipoprotein (HDL)–cholesterol analysis was carried out on an Olympus 640 analyzer using the direct method (no precipitation). Low HDL-cholesterol was defined as values <1.0 mmol/L for men or <1.3 mmol/L for women and/or taking cholesterol management medication (13). Total glycated hemoglobin (HbA1c) assay was measured using the Tosoh G7 analyzer. Elevated HbA1c was defined as values above 48 mmol/mol. Diabetes was defined as elevated HbA1c and/or medication and/or self-reported diabetes. Respiratory function was based on forced expiratory volume (FEV1), defined as the amount of air (in liters) that a participant could exhale with maximal effort during the first second following full inspiration. FEV1 was then expressed in percentage of the predicted normal for a person of the same sex, age, and height; we used the threshold of 80 per cent to define suboptimal pulmonary function (14).

### Assessment of Depressive Symptoms and Memory Impairment

Depressive symptoms were ascertained using the 8-item Center for Epidemiologic Studies Depressive (CES-D) scale (15). Presence of depressive symptoms was defined as having a score of 4 or above (range 0 to 8) (16) and/or self-reporting antidepressant medication use. A memory score, our marker of cognitive function, was based on the summation of immediate and delayed recall results from a 10-word list–learning test (score range: 0 to 20). Memory impairment was defined as belonging to the lowest sex and age-specific quintile of memory score. Depressive symptoms and memory score at wave 1 were used as baseline covariates in the respective models.

### Definition of Healthy Aging

Based on an existing approach (17), we computed a composite “healthy aging” index defined as an absence of chronic disease (coronary heart disease, stroke, diabetes, and cancer), disability (reported limitation in at least one ADL or IADL), depressive symptoms (CES-D score ≥ 4), and more favorable levels of memory, respiratory function, blood pressure, and walking speed—the latter four defined as being in the top 4 sex- and age-specific quintiles.

### Statistical Analysis

Group-based trajectories of CRP were derived using latent class growth mixture modeling with Proc TRAJ (SAS 9.4, SAS Institute, Cary, NC) (18). This data-driven approach allows individuals with similar trajectories to be grouped (19), returning trajectories of average values within homogenous subgroups, while not capturing the within-person variability. We selected the best model fit among a finite set of models including up to five classes (trajectories) with both linear and quadratic terms in each class. CRP was used in its original scale, since after truncation at 10 mg/L its distribution approximated normality. The best fit was determined based on the following criteria: lower Bayesian Information Criterion (BIC); mean posterior probabilities of group assignment (ie the likelihood of an individual assigned to a class to belonging to the given class) ≥ 70 per cent; every class containing at least 5 per cent of the participants; and distinction between the classes. When the best unadjusted model was ascertained, estimates were then adjusted for sex and baseline age, BMI (continuous), smoking status (current, other), education (three levels), physical activity level (four levels), and use of anti-inflammatory drugs (yes/no). Models of healthy aging and of physical functioning tests (balance, chair rise, grip test, and walking speed) also included adjustment for baseline arthritis (measured at wave 1). When available, baseline measurements of the specific outcomes were included in the models as covariates, namely, as follows: baseline number of limitations with ADL or IADL when the outcome was disability with ADL or IADL, respectively, baseline blood pressure lowering medication when the outcome was hypertension, baseline depressive symptoms when the outcome was depression, and baseline memory score when the outcome was memory impairment. As main analyses, multivariable logistic regression models were fitted to produce odds ratios (ORs) with accompanying 95% confidence interval (95% CI) for the association of CRP trajectories with each of the aging outcome.

To assess the potential added value of monitoring long-term change of inflammation, we also assessed in cross-sectional analyses the associations between final levels of CRP (measured at wave 6) with concomitant aging outcomes, to compare the significance and magnitude of estimates. We categorized CRP levels as <3 vs ≥3 mg/L, which is a threshold used to define the presence of systemic inflammation (20).

We performed three sets of sensitivity analyses. In the first set, we assessed incidence of new onset of impairment by excluding individuals with the condition at baseline, but this was only possible for limitations in ADL and IADL, arthritis, hypertension, low HDL–cholesterol, obesity, depressive symptoms, and memory impairment (no available clinical test for balance, chair rise, grip strength, walking speed, and respiratory function before wave 2). In the second set of analyses, we tested whether the results led to the same conclusions when using Poisson regression with robust error variance for estimation of relative risks for frequent outcomes defined as prevalence at wave 6 > 20 per cent (21): healthy aging (35 per cent), balance impairment (24 per cent), obesity (27.5 per cent), and hypertension (51.2 per cent). Finally, in the third set of analyses, we used generalized linear models (GLMs) to assess the association between CRP trajectories and aging phenotypes that were coded on a continuous scale: BMI, BP, blood biomarkers, FEV1, grip strength, walking speed, number of difficulties with ADL and IADL, number of depressive symptoms, and memory score.

**Figure 1.**
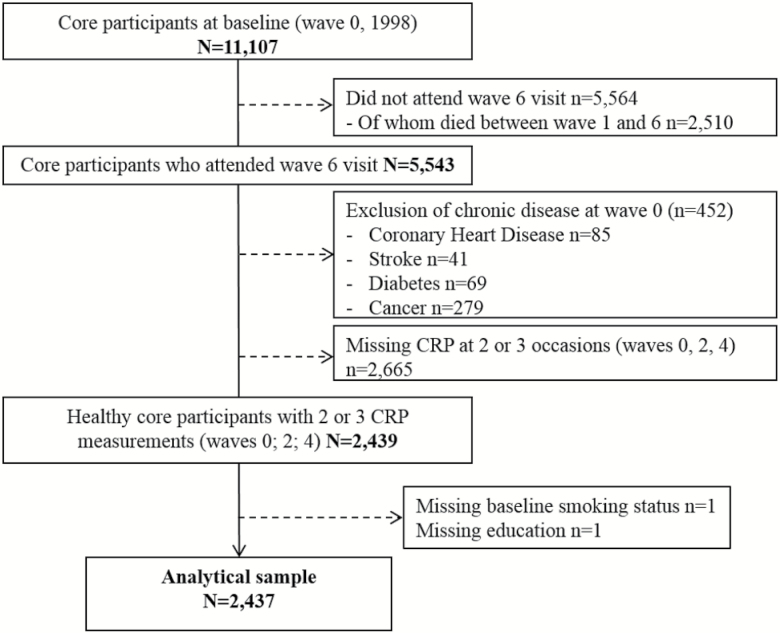
Flow chart of the selection of the analytical sample, the English Longitudinal Study of Ageing.

To be included in the present analyses, participants had to have at least two assessments of CRP (<10 mg/L) over three nurse visits (wave 0, 1998–2001; wave 2, 2004–2005; wave 4, 2008–2009) and have attended the wave 6 (2012–2013) interview and nurse examination for the assessment of aging outcomes. By excluding participants with baseline self-reported physician–diagnosed chronic disease (coronary heart disease, stroke, diabetes, and cancer), our analyses are restricted to a healthy group of 2,437 men and women—our analytical sample. The selection of participants is presented in Figure 1. The selection criteria of the analytical sample involved exclusion of a large number of participants who did not have at least two measurements and did not attend the wave 6 visit. Therefore, we compared baseline characteristics of those included in the analyses with those excluded and those who died during follow-up. The differences were formally tested across the three groups using ANOVA for continuous variables and chi-square tests for categorical variables.

### Patient Involvement

This paper is based on a longitudinal cohort study in which we recruited members of the general population and collected data on an array of potential risk factors and health outcomes. These data are freely available to the research community. As a study of the general population, participants were not involved in setting the research question or the outcome measures, nor were they involved in developing plans for design or implementation of the study. No participants were asked to advise on interpretation or writing up of results.

## Results

**Figure 2.**
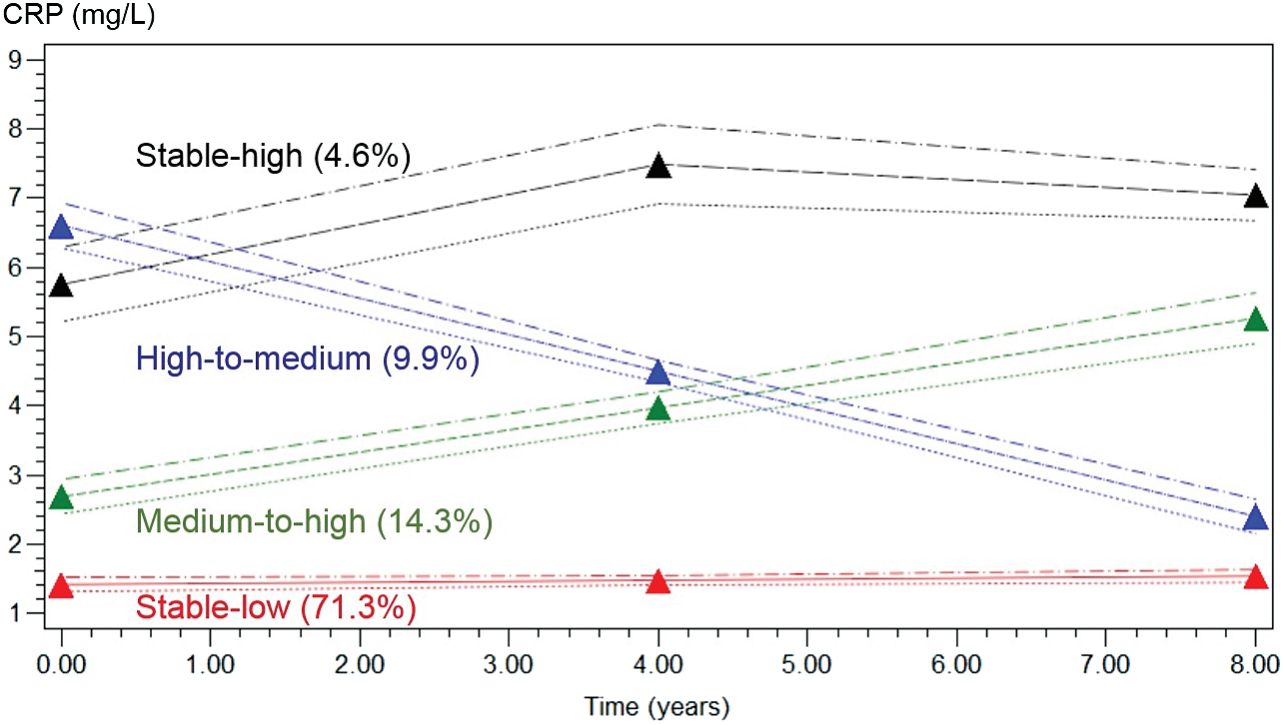
Adjusted trajectories^a^ of C-reactive protein (mg/L) and their 95% confidence intervals over the period 1998–2009, the English Longitudinal Study of Ageing. Number in parenthesis are the percentage of the sample included in each group trajectory. ^a^Trajectories are adjusted for sex and baseline age, body mass index, smoking status, physical activity level, educational level, and use of anti-inflammatory drugs.

Four CRP trajectories (classes) were identified, and the mean predicted trajectory in each class is presented in Figure 2. A “stable-low” trajectory with a baseline mean CRP value of 1.33 mg/L and a linear slope of 0.02 (*p* = .01) included 71 per cent of the population and is used hereafter as the reference category. The second class was represented by a “medium-to-high” trajectory (intercept 2.7 and linear slope 0.32, *p* < .001) and included 14 per cent of the sample; the third class, the “high-to-medium” trajectory (intercept 6.6 mg/L, slope −0.53, *p* < .001), comprised 10 per cent of the sample; the fourth class, the “stable-high” trajectory (intercept 5.7, linear slope 0.71, quadratic term −0.07, all *p* < .001) included 5 per cent of the sample. Of note, the medium-to-high trajectory displayed a baseline value lower than the 3.0 mg/L threshold to define the presence of inflammation, and in the high-to-medium trajectory, the CRP levels at the end of the time period are below the 3.0 mg/L threshold.

Baseline characteristics (1998–2001) according to each of these classes of trajectory are described in Table 1. Compared with individuals following a stable-low trajectory, participants belonging to the other classes were generally older, more likely to be smokers, less well educated, less physically active, and had a higher BMI, although this was not always the case for the stable-high trajectory. They were also more likely to have arthritis, difficulties with ADL and IADL, depression, and had a worse cardiometabolic profile. Overall during follow-up, there was an increase in BMI, use of antihypertensive drugs, in disability and arthritis prevalence, whereas there was a decrease in smoking and depressive symptoms. Of the 11,107 participants present at baseline (wave 0), 8,669 were excluded from our analysis (of whom 2,510 died by wave 6). Compared with the analytical sample, participants who were excluded were older, had higher baseline CRP values and lower education, and were more likely to be current smokers, to have hypertension, to be less physically active, to have depressive symptoms, and to report limitations with ADL. Participants who died during follow-up exhibited a less favorable profile than the excluded ones (Supplemental Table 1).

**Table 1.** Baseline (wave 0, 1998–2001) and End of Follow-up (wave 6, 2012/2013) Characteristics According to Each CRP Trajectory in the English Longitudinal Study of Aging

		CRP Trajectory			
	All	Stable-Low	Medium-to-High	High-to-Medium	Stable-High
	Baseline				
*n*	2437	1762	337	231	107
Sex, % female	56.5	54.5	57.6	67.1	64.5
Age, y, mean ± SD	58.8 ± 8.1	58.4 ± 7.9	59.3 ± 8.5	61.1 ± 8.3	58.8 ± 7.6
BMI (kg/m^2^)	27.1 ± 4.1	26.3 ± 3.6	28.7 ± 4.6	29.2 ± 4.8	30.6 ± 4.8
Number of CRP occasions	2.3 ± 0.5	2.4 ± 0.5	2.3 ± 0.5	2.3 ± 0.5	2.3 ± 0.5
Education, %					
Low	30.7	26.9	37.7	45.9	38.3
Medium	39.6	40.3	40.9	32.5	38.3
High	29.7	32.8	21.4	21.6	23.4
Current smokers, %	14.7	13.3	18.7	17.7	18.7
Use of NSAID, %	12.7	11.0	14.5	20.3	17.8
Use of antihypertensive drug, %	13.2	11.2	18.7	19.0	15.9
Vigorous physical activity, %	22.4	25.3	19.1	10.3	13.7
Arthritis^†^, %	26.5	24.3	27.4	36.4	38.3
Limitation with ADL^†^, %	10.1	7.4	14.2	18.6	23.4
Limitation with IADL^†^, %	9.2	7.8	11.9	14.7	12.2
Depression (CES-D ≥ 4)^†^, %	10.8	9.7	11.5	15.4	17.0
Glycated hemoglobin^‡^ (%)	5.47 ± 0.52	5.44 ± 0.49	5.58 ± 0.64	5.53 ± 0.45	5.61 ± 0.63
HDL-cholesterol^‡^ (mmol/L)	1.57 ± 0.39	1.6 ± 0.39	1.48 ± 0.36	1.5 ± 0.37	1.47 ± 0.35
Total cholesterol^‡^ (mmol/L)	6.07 ± 1.17	6.06 ± 1.15	6.01 ± 1.1	6.18 ± 1.35	6.14 ± 1.29
Systolic blood pressure^‡^ (mm Hg)	133.0 ± 17.3	131.7 ± 17.1	135.7 ± 16.9	137.5 ± 18.8	135.1 ± 14.9
	End of follow-up				
Age, y	71.3 ± 7.8	70.9 ± 7.6	72.0 ± 8.3	73.6 ± 7.8	71.5 ± 7.6
BMI (kg/m^2^)	27.7 ± 4.7	26.8 ± 4.1	30.1 ± 5.2	29.4 ± 4.7	31.8 ± 5.7
Current smokers, %	8.0	7.1	11.0	9.5	11.2
Use of antihypertensive drug, %	28.5	25.5	38.0	36.4	31.8
Vigorous physical activity, %	20.6	23.2	16.6	10.4	12.2
Arthritis, %	42.6	39.6	51.9	47.2	54.2
Limitation with ADL, %	15.5	11.9	27.7	21.7	22.4
Limitation with IADL, %	13.1	10.8	19.9	20.4	14.0
Depression (CES-D ≥ 4), %	9.5	8.0	14.1	13.6	9.4
Glycated haemoglobin (%)	5.88 ± 0.65	5.82 ± 0.54	6.06 ± 0.94	5.94 ± 0.67	6.15 ± 0.89
HDL-cholesterol (mmol/L)	1.69 ± 0.48	1.73 ± 0.48	1.57 ± 0.47	1.61 ± 0.42	1.55 ± 0.46
Total cholesterol (mmol/L)	5.51 ± 1.18	5.56 ± 1.2	5.42 ± 1.17	5.37 ± 1.06	5.28 ± 1.08
Systolic blood pressure (mm Hg)	133.3 ± 17.7	132.4 ± 17.2	135.6 ± 18.9	135.5 ± 18.2	135.1 ± 18.9
Incident cancer, %	5.3	5.1	5.9	6.1	6.5
Incident CHD, %	9.7	8.6	11.0	16.0	9.4
Incident stroke, %	4.2	3.8	5.6	6.1	1.9
Incident diabetes, %	10.0	8.0	15.1	13.4	19.6

Notes: ^†^Measured at wave 1 (2002/2003); ^‡^measured at wave 2 (2004/2005).

CRP = C-reactive protein; ADL = Activities of daily living; IADL = Instrumental ADL; NSAID = Non-steroidal anti-inflammatory drugs.

**Figure 3.**
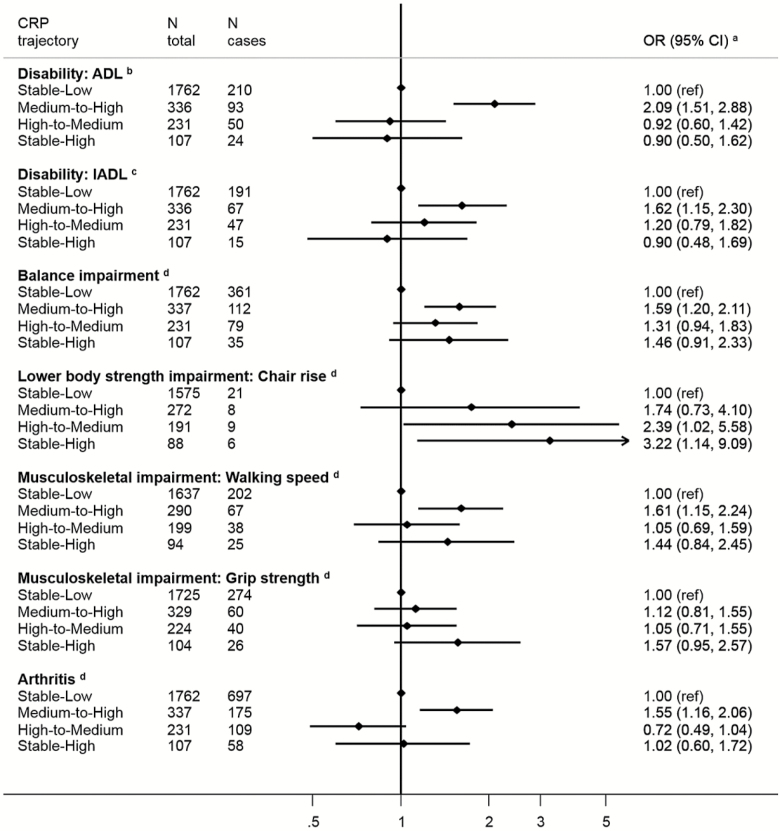
Odds ratios (OR; 95% CI) for the associations between C-reactive protein trajectories and impairment in physical functioning at wave 6 (2012–2013), the English Longitudinal Study of Aging. ^a^All ORs are adjusted for sex and baseline age, body mass index, smoking status, physical activity level, educational level, and use of anti-inflammatory drugs. ^b^ORs are further adjusted for baseline number of activities of daily living (ADL) difficulties (reported at wave 1). ^c^ORs are further adjusted for baseline number of instrumental ADL difficulties (reported at wave 1). ^d^ORs are further adjusted for baseline arthritis (reported at wave 1).

**Figure 4.**
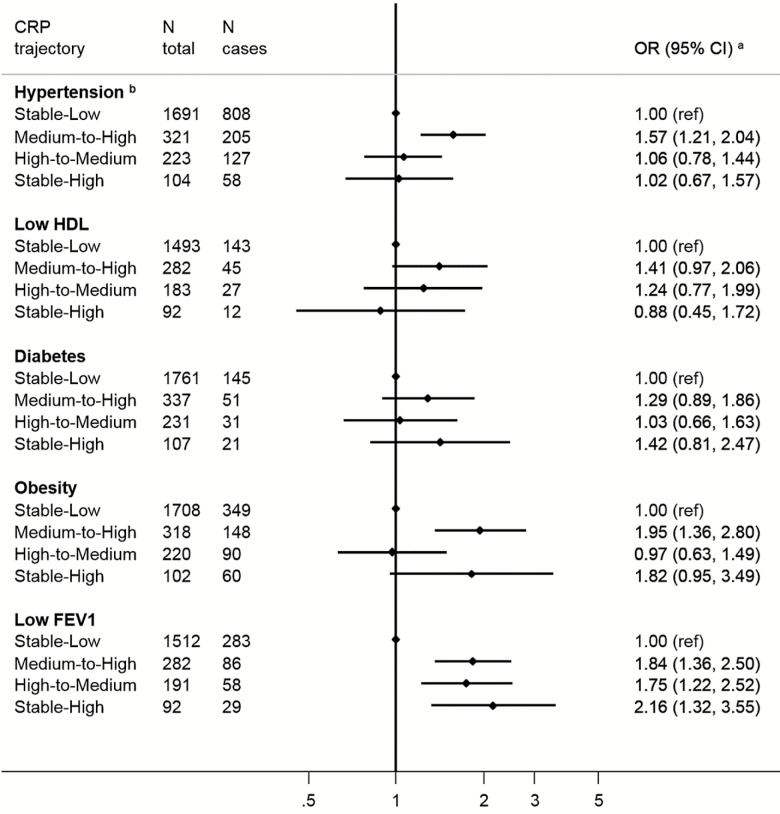
Odds ratios (OR; 95% CI) for the associations between C-reactive protein trajectories and cardiometabolic markers and impairment in respiratory functioning at wave 6 (2012–2013), the English Longitudinal Study of Ageing. ^a^All ORs are adjusted for sex and baseline age, body mass index, smoking status, physical activity level, educational level, and use of anti-inflammatory drugs. ^b^ORs are further adjusted for baseline use of antihypertensive medication.

**Figure 5.**
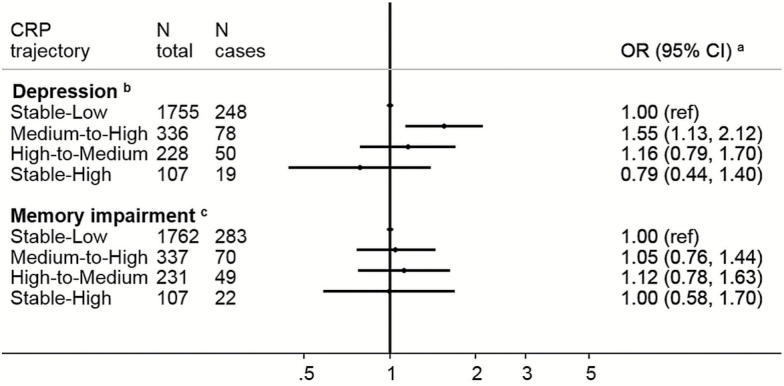
Odds ratios (OR; 95% CI) for the associations between C-reactive protein trajectories and mental health outcomes at wave 6 (2012–2013), the English Longitudinal Study of Ageing. ^a^All ORs are adjusted for sex and baseline age, body mass index, smoking status, physical activity level, educational level, and use of anti-inflammatory drugs. ^b^ORs are further adjusted for baseline depressive symptoms (reported at wave 1). ^c^ORs are further adjusted for baseline memory score (reported at wave 1).

Next, in longitudinal analyses, we related these CRP trajectories to the aging end points. Relative to the stable-low group (our referent), membership of the medium-to-high trajectory was associated with higher odds of subsequent disability (ADL: OR; 95% CI: 2.09; 1.51; 2.88; IADL: 1.62; 1.15; 2.30), balance impairment (1.59; 1.20, 2.11), walking speed impairment (1.61; 1.15, 2.24), and arthritis (1.55; 1.16, 2.06; Figure 3). The two other trajectories were only significantly associated with increased odds of lower body strength (high to medium OR = 2.39; 1.02, 5.58 and stable high 3.22; 1.14, 9.09). Finally, belonging to the stable-high trajectory was associated with low grip strength, walking speed, and balance impairment but precision of the estimates was low and they did not reach statistical significance at the conventional level. For cardiometabolic and respiratory functioning outcomes, the medium-to-high trajectory was also the class most consistently associated with increased odds of subsequent diabetes, hypertension and less favorable levels of HDL-cholesterol, and BMI (Figure 4). All three nonreference trajectories showed positive associations with low respiratory functioning. Belonging to the stable-high was associated with diabetes and obesity but not significantly. Belonging to the medium-to-high CRP trajectory was associated with higher odds of depression (OR = 1.55; 1.13, 2.12). No significant trend was observed with memory impairment (Figure 5).

Of 1,886 participants with all available data, 663 were categorized as having a healthy aging phenotype at wave 6. Compared with people in the stable-low trajectory, participants belonging to the medium-to-high (OR = 0.57; 0.40, 0.79) and stable-high (OR = 0.50; 0.27, 0.91) CRP trajectories displayed lower odds of healthy aging. The high-to-medium trajectory was not significantly associated with healthy aging (OR = 0.86; 0.58, 1.26).

In cross-sectional analyses, we found a strong inverse association between final levels of CRP and healthy aging (OR _elevated vs normal_ = 0.54; 0.40, 0.72). When focusing on individual measures of impairment (Supplementary Figure 1), there were statistically significant cross-sectional associations with IADL disability, musculoskeletal impairment, obesity, and depressive symptoms.

### Sensitivity Analyses

When individuals with pre-existing condition were excluded, the associations with subsequent disability with ADL, IADL, and with arthritis remained virtually unchanged, with only the medium-to-high trajectory displaying higher odds of impairment (Supplementary Figure 2). Similarly, after exclusion of participants with hypertension or obesity, OR estimates were comparable with the main analysis. The associations between the medium-to-high trajectory and low HDL-cholesterol and depression were attenuated after exclusion of participants with the condition at baseline (Supplementary Figures 3 and 4). When we modeled the relative risks from Poisson regression for nonrare outcomes, the conclusions remained unchanged, with similar direction and significance of the estimates to the ORs obtained from logistic regression, only the magnitude was lower (Supplementary Figure 5). Finally, the linear associations of CRP trajectories with continuously scored outcomes displayed generally the same pattern as those seen using logistic regressions (Supplementary Table 2). Consistently with the logistic regression analysis, the most at-risk trajectory identified was medium-to-high which was associated with higher SBP, HbA1c, BMI, lower HDL-cholesterol, FEV1, more difficulties with ADL, and more depressive symptoms. The high-to-medium trajectory displayed associations with lower HDL-cholesterol and FEV1, whereas the stable-high trajectory was associated with higher HbA1c, lower HDL-cholesterol, lower FEV1, and lower grip strength.

## Discussion

In a population-based sample of English older adults, we identified four long-term trajectories of CRP over a 10 year period and related these trajectories to various components of a healthy aging phenotype. Our main finding was that, independently of health behaviors, socioeconomic status, BMI, and use of anti-inflammatory drugs, increasing CRP from medium to high levels was associated with subsequent poor cardiometabolic health, lower physical and respiratory functioning, and increased depressive symptoms and arthritis. Maintaining elevated levels (“stable-high”) was also associated with some impaired aging phenotypes. The odds of composite healthy aging index were 43% to 50% lower for participants belonging to medium-to-high or stable-high CRP trajectories compared with those with a stable-low CRP trajectory.

The link between inflammatory markers and disability, impaired musculoskeletal functioning, osteoarthritis, and, most broadly, with frailty has been the object of various studies (22–28). However, the vast majority has been of cross-sectional design and the few prospective studies had only one measurement of CRP and therefore were not able to assess the predictive capacity of change in inflammation status. Most studies found that higher levels of CRP were associated with lower grip strength, walking speed, and more difficulties with ADL (22–27). In the Cardiovascular Health Study All Stars, where CRP was measured at two time points 9 years apart, doubling of CRP was associated with 18 per cent increased odds of concurrent onset of new physical impairment with ADL, walking speed, and grip strength (29). Our results provide novel insight into a more comprehensive set of physical functioning domains. We showed that a gradual increase in CRP over almost 10 years was associated with subsequent impairment in balance, walking speed, lower body strength, and ADL- and IADL-related disability and arthritis, after taking into account arthritis and disability at baseline. Maintaining elevated levels of CRP was also associated with some of these domains but to a lesser extent, indicating that increasing levels may confer greater risk than persistently high levels.

The role of inflammation in atherosclerosis and cardiovascular dysfunction is well documented (30), even if a potential direct causal role of CRP on coronary disease is unlikely in the light of recent Mendelian Randomization studies (31), whereas other markers involved in the inflammatory pathways are more likely to be causal, such as IL-6 (32). Hence, there is still debate whether CRP is a risk factor or a risk marker of the inflammatory activity occurring in the atherosclerotic lesions, but despite this, CRP is a potentially useful marker for the prediction of cardiovascular risk (33). A meta-analysis of mostly cross-sectional studies relating CRP to cardiovascular disease risk factors showed strong correlations between elevated levels of CRP and diabetes, hypertension, and dyslipidemia (8). Our study provides a unique description of long-term CRP trajectories in early old age in relation to adverse cardiometabolic outcomes. We showed that a steady increase in CRP over time was associated with subsequent hypertension, low HDL-cholesterol, and obesity, and maintaining high levels of CRP was associated with diabetes and obesity.

We found strong associations with a marker of respiratory function (FEV1), showing that unfavorable CRP trajectories were related to increased risk of poorer respiratory function. A few studies have investigated the association between CRP and respiratory function prospectively and are in accordance with our results: analyses in the EPIC-Norfolk cohort showed that changes in levels of serum CRP between baseline and 13 year follow-up (two measurements) were negatively associated with changes in FEV1, whereas baseline CRP alone was not associated with the change (34) and stronger negative association with number of high IL-6 occasions than only with baseline IL-6, and FEV1 was found in the Whitehall II cohort (17). The possible mechanisms by which systemic inflammation can damage lung tissue are senescence of lung alveolar and endothelial cells via telomere shortening and pulmonary vascular filtration due to endothelial dysfunction (35).

Studies on cognitive functioning and decline are more inconclusive and only a weak overall association with inflammation has been suggested from the literature (36). The same limitation that most studies are cross-sectional or single-measurement longitudinal studies applies, and we are only aware of three studies with two measurements. In the CHS All Stars (29), doubling of CRP was associated with cognitive impairment assessed by Mini Mental State Examination (MMSE) and digit symbol substitution test score, but in the Whitehall II study (17), high levels of IL-6 at one or two occasions over a 5 year exposure period were not significantly associated with lower odds of good cognitive functioning. In the Epidemiology of Hearing Loss Study, maintaining high CRP levels over 10 years (two measurements) was even associated with lower risk of cognitive impairment compared with maintaining low levels, whereas a doubling of CRP levels over 20 years was associated with higher risk but only in statin nonusers (37). We did not observe any significant association with memory impairment in the present study. Inflammation has been however more consistently associated with risk of dementia (38), suggesting markers such as CRP may be better predictors for later stage of neurodegeneration but do not have great predictive value for mild cognitive impairment.

An overall weak but significant effect of CRP levels on depressive symptoms in longitudinal studies has been described and meta-analyzed (39) even if some individual studies, including ELSA, showed no significant association with baseline CRP and subsequent depressive symptoms (40). The results of the present analysis provide further insight and show that relatively low baseline CRP but increasing over time is associated with subsequent depression. They come to complement a recent study which found that repeated exposure to systemic inflammation was associated with higher odds of depression (41).

Finally, in accordance with our results, two studies have shown that repeatedly high or increase over time in CRP values has been associated with lower odds of successful aging (17) or higher odds of functional (physical and cognitive) impairment (29). The present findings highlight the importance of CRP as a simply assessed biomarker of inflammation and predictor of (un)healthy aging. Overall, an increase from normal to high levels of CRP and constant high levels seemed to confer greater risk of adverse aging outcomes, compared with unstable but declining trajectories.

When comparing the trajectories to final levels of CRP, our results are similar to the ones by Jenny and colleagues in the CHS All Stars study (29), who report a strong association of any impairment or physical impairment, both with final levels of CRP and for 9 year change. However, there was no association with cognitive impairment in our study, whereas Jenny and colleagues reported an association with CRP change, but not with final levels. The individual associations were overall more marked with the trajectory analysis (ORs for medium-to-high trajectory) than with final levels.

### Study Strengths and Limitations

The main strength of this study is that it is a large, nationally representative sample of older people living in England with long-term follow-up. The repeat measurement of serum CRP levels is a rare feature that allowed the description of trajectories of inflammation over 10 years and longitudinal associations with aging outcomes. Furthermore, modeling CRP as a continuous variable allowed a more detailed description of the trajectories over time than a categorization as above or below a clinical threshold. If a single baseline measurement would have been used, we would not have been able to identify the medium-to-high trajectory as an “at-risk” group. The physical functioning and cardiometabolic outcomes were objectively measured during a nurse visit using standardized procedures, limiting the risk of misclassification bias.

The main limitation is that the analytical sample was only 21 per cent of the original ELSA sample. This resulted in a sample of relatively younger and healthier individuals. Our estimates should be interpreted in the light of this limitation, as outcomes were only assessed in participants who were able to attend wave 6 visit and who had at least two CRP measurements. The selective loss to follow-up of vulnerable participants and inclusion of healthier participants (known as the “healthy survivor effect”) may explain the absence of association for the stable-high trajectory with outcomes such as disability, arthritis, or depression, as this group was very small and younger than the other at-risk trajectories. In contrast, participants in the high-to-medium trajectory were older but overall that group showed a healthier lifestyle and a somewhat stabilized or improved cardiometabolic profile at the end of the follow-up, which may partly explain the shape of the CRP trajectory and the lack of association with aging outcomes. Another implication of the sample selection is that our findings may not be generalizable to older, less healthy population. A second limitation is that we selected generally healthy participants at baseline by excluding participants with cardiovascular disease, diabetes, and cancer, but to avoid further exclusions in the main analysis, we did not exclude but rather adjusted for the presence of disability, arthritis, antihypertensive medication, depression, or cognitive function when modeling respective subsequent outcomes. Therefore, we did not strictly assess incidence of new aging outcomes. However, when possible, in sensitivity analyses, we did exclude baseline prevalent cases of impairment, which lead to substantially similar results. Furthermore, we tried to address confounding by subclinical cardiovascular disease (atherosclerosis), associated with both inflammation and disability, by excluding baseline cardiovascular disease and diabetes, and by adjusting for BMI and antihypertensive drugs. However, we cannot rule out the existence of residual confounding by unmeasured differences in atherosclerosis between the participants. Moreover, the relationship between CRP trajectories and subsequent outcomes may have been mediated by incident chronic conditions such as coronary heart disease, stroke, or cancer occurring during the follow-up, which can have strong deleterious physical and mental health consequences. But when repeating analyses including incident chronic disease in the models, results remained unchanged except for IADL and walking speed for which ORs were slightly attenuated but remained significant. Lastly, latent class growth mixture modeling is a data-driven technique and as such limits comparability with other studies and generalizability of the findings. Finally, some of our outcomes (disability assessed through limitations in ADL and IADL, depressive symptoms) and covariates (smoking, physical activity, and drug use) were self-reported, which can be prone to measurement error and can bias the results.

To conclude, in this population-based study of English older adults, we were able to describe inflammation trajectories over a 10 year period and showed that an increase in CRP or repeatedly high levels of CRP was associated with various adverse aging outcomes, considered separately or as a composite phenotype. Further research should focus on assessing whether individual-level trajectories can predict aging outcomes to potentially guide the development of risk prediction algorithms including repeated CRP values.

## Authors’ Contributions

C.L., P.Z., and G.D.B. generated the idea for the present study. C.L. conducted all analyses, interpreted the results, and drafted the manuscript. G.D.B., P.Z., D.C., T.N.A., M.K., and A.S. were responsible for interpreting the results and critically revising the manuscript. All authors have read and approved the final manuscript.

## Funding

The English Longitudinal Study of Ageing is supported by the National Institute on Aging (grant numbers: 2RO1AG7644 and 2RO1AG017644-01A1) and a consortium of the UK government departments coordinated by the Economic and Social Research Council. M.K. is supported by the Medical Research Council (K013351), NordForsk, and the Academy of Finland (311492). The funding bodies had no role in the study design; in the collection, analysis, and interpretation of data; in the writing of the manuscript; and in the decision to submit the manuscript for publication.

## Conflict of Interest

C.L. affirms that the manuscript is an honest, accurate, and transparent account of the study being reported; that no important aspects of the study have been omitted; and that any discrepancies from the study as planned (and, if relevant, registered) have been explained. ELSA data is open access at https://www.ukdataservice.ac.uk/

## Supplementary Material

Supplemental Tables

Supplemental Figures
