# Supplementary material for: Association of 10-Year C-Reactive Protein Trajectories With Markers of Healthy Aging: Findings From the English Longitudinal Study of Aging
Source: J Gerontol A Biol Sci Med Sci. 2018 Feb 15;74(2):195–203. doi: 10.1093/gerona/gly028 (PMC6333942; doi:10.1093/gerona/gly028)
Supplement: Supplemental Tables [file gly028_suppl_supplemental_tables.docx]

**Supplemental Table 1.** Comparison of baseline characteristics of participants included and excluded from the analytical sample

|  | Included | Excluded | Died by wave 6 | p difference | Included with no missing | Excluded with no missing | Dead with no missing |
| --- | --- | --- | --- | --- | --- | --- | --- |
| n | 2437 | 6119 | 2550 |  |  |  |  |
| Sex, % female | 56.5 | 56.88 | 48.2 | <.0001 |  |  |  |
| Age, years | 58.8 ±8.1 | 60.9 ±9 | 71.9 ±9.5 | <.0001 | 2437 | 6119 | 2550 |
| BMI, kg/m2 | 27.1 ±4.1 | 27.9 ±4.7 | 27.5 ±4.7 | <.0001 | 2437 | 5450 | 2154 |
| CRP, mg/L | 2.27 ±2.11 | 2.51 ±2.18 | 3.02 ±2.27 | <.0001 | 1229 | 1650 | 815 |
| Number of CRP occasions | 2.34 ±0.47 | 0.72 ±0.79 | 0.59 ±0.74 | <.0001 | 2437 | 6119 | 2550 |
| Education, % |  |  |  | <.0001 | 2437 | 6117 | 2549 |
| Low | 30.7 | 43.67 | 58.14 |  |  |  |  |
| Medium | 39.6 | 35.08 | 27.81 |  |  |  |  |
| High | 29.8 | 21.25 | 14.04 |  |  |  |  |
| Current smokers, % | 14.7 | 20.27 | 21.65 | <.0001 | 2437 | 6117 | 2550 |
| Use of NSAID, % | 12.68 | 17.43 | 32 | <.0001 | 2437 | 6119 | 2550 |
| Use of antihypertensive drug, % | 13.21 | 19.35 | 34.43 | <.0001 | 2437 | 6119 | 2550 |
| Hypertensive, % | 41.5 | 51.1 | 63.2 | <.0001 | 1943 | 4005 | 1768 |
| Vigorous physical activity, % | 22.4 | 13.28 | 5.11 | <.0001 | 1619 | 3606 | 1624 |
| Arthritis wave 1, % | 26.5 | 32.38 | 40.42 | <.0001 | 2436 | 6115 | 2548 |
| Limitation with ADL wave 1, % | 10.1 | 18.21 | 37.57 | <.0001 | 2437 | 6045 | 2489 |
| Limitation with IADL wave 1, % | 9.2 | 18.88 | 40.26 | <.0001 | 2437 | 6045 | 2489 |
| Depression wave 1 (CES-D>=4), % | 10.8 | 16.01 | 22.91 | <.0001 | 2407 | 5907 | 2396 |

**Supplemental Table 2.** Generalized linear regression models for associations between CRP trajectories and continuous outcomes at wave 6 (2012-2013), the English Longitudinal Study of Ageing

|  |  | CRP trajectory | | | | | | |
| --- | --- | --- | --- | --- | --- | --- | --- | --- |
|  |  | Stable-low | Medium-to-high | | High-to-medium | | Stable-high | |
| **Continuous outcome** | **N total** | B (SE) ^a^ | B (SE) ^a^ | p-value | B (SE) ^a^ | p-value | B (SE) ^a^ | p-value |
| **Cardiometabolic health** | |  |  |  |  |  |  |  |
| SBP (mm Hg) ^b^ | 2303 | Ref | 2.52 (1.11) * | 0.02 | 2.24 (1.31) | 0.09 | 1.76 (1.84) | 0.34 |
| HDL-cholesterol (mmol/L) | 2038 | Ref | -0.1 (0.03) * | 0.001 | -0.09 (0.03) * | 0.01 | -0.09 (0.05) * | 0.07 |
| HbA1c (mmol/mol) | 2019 | Ref | 1.70 (0.46) * | 0.0002 | 0.16 (0.57) | 0.78 | 2.02 (0.77) * | 0.01 |
| BMI (kg/m2) | 2348 | Ref | 0.95 (0.15) * | <.0001 | -0.06 (0.18) | 0.74 | 0.74 (0.26) * | 0.004 |
| **Respiratory function** |  |  |  |  |  |  |  |  |
| FEV1 (% predicted value) | 2077 | Ref | -5.57 (1.61) * | 0.001 | -5.39 (1.92) * | 0.01 | -6.2 (2.72) * | 0.02 |
| **Physical functioning** |  |  |  |  |  |  |  |  |
| Grip strength (kg) ^c^ | 2381 | Ref | 0.07 (0.41) | 0.87 | -0.31 (0.49) | 0.53 | -1.92 (0.69) * | 0.01 |
| Walking speed (m/s) ^c^ | 2219 | Ref | -0.02 (0.01) | 0.26 | 0 (0.02) | 0.85 | -0.01 (0.02) | 0.64 |
| ADL (number of difficulties) ^d^ | 2436 | Ref | 0.14 (0.05) * | 0.003 | -0.05 (0.05) | 0.35 | -0.02 (0.08) | 0.75 |
| IADL (number of difficulties) ^e^ | 2436 | Ref | 0.05 (0.04) | 0.16 | 0 (0.04) | 0.91 | -0.03 (0.06) | 0.66 |
| **Mental health** |  |  |  |  |  |  |  |  |
| Depressive symptoms (0-8) ^f^ | 2422 | Ref | 0.26 (0.09) * | 0.01 | 0.09 (0.11) | 0.45 | -0.05 (0.16) | 0.73 |
| Memory score (0-20) ^g^ | 2437 | Ref | -0.11 (0.18) | 0.56 | -0.19 (0.22) | 0.38 | 0.09 (0.3) | 0.77 |

^a^ Regression coefficients adjusted for sex and baseline age, body mass index, smoking status, physical activity level, educational level and use of anti-inflammatory drugs; ^b^ Further adjusted for baseline antihypertensive drug use; ^c^ Further adjusted for baseline arthritis; ^d^ Further adjusted for baseline ADL difficulties; ^e^ Further adjusted for baseline IADL difficulties; ^f^ Further adjusted for baseline depressive symptoms; ^g^ Further adjusted for baseline memory score. * P<0.05

Abbreviations: SBP, systolic blood pressure; HbA1c, glycated haemoglobin; HDL-chol, high density lipoprotein cholesterol; BMI, body mass index; FEV1, forced expiratory volume in 1 second; ADL, activities of daily living; IADL; instrumental activities of daily living; CES-D, centre for epidemiologic studies depressive scale.
