# Supplementary material for: Association of 10-Year C-Reactive Protein Trajectories With Markers of Healthy Aging: Findings From the English Longitudinal Study of Aging
Source: J Gerontol A Biol Sci Med Sci. 2018 Feb 15;74(2):195–203. doi: 10.1093/gerona/gly028 (PMC6333942; doi:10.1093/gerona/gly028)
Supplement: Supplemental Figures [file gly028_suppl_supplemental_figures.docx]

**Supplemental Figure 1.** Cross-sectional associations (OR and 95% CI) between elevated CRP (≥3 vs <3 mg/L) and ageing outcomes at wave 6 (2012-2013), the English Longitudinal Study of Ageing

**
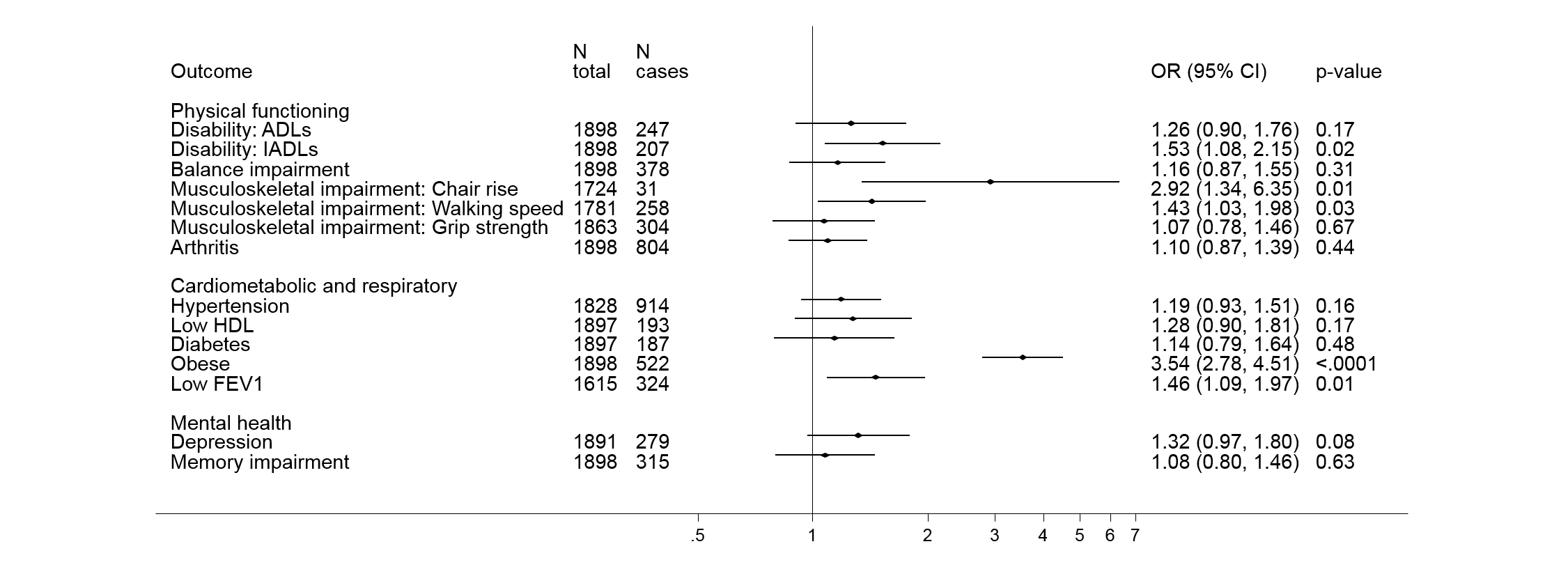
**

**Supplemental Figure 2**. Odds ratios (95% CI) for the associations between CRP trajectories and impairment in physical functioning at wave 6 (2012-2013) after excluding participants with respective impairment at baseline, the English Longitudinal Study of Ageing


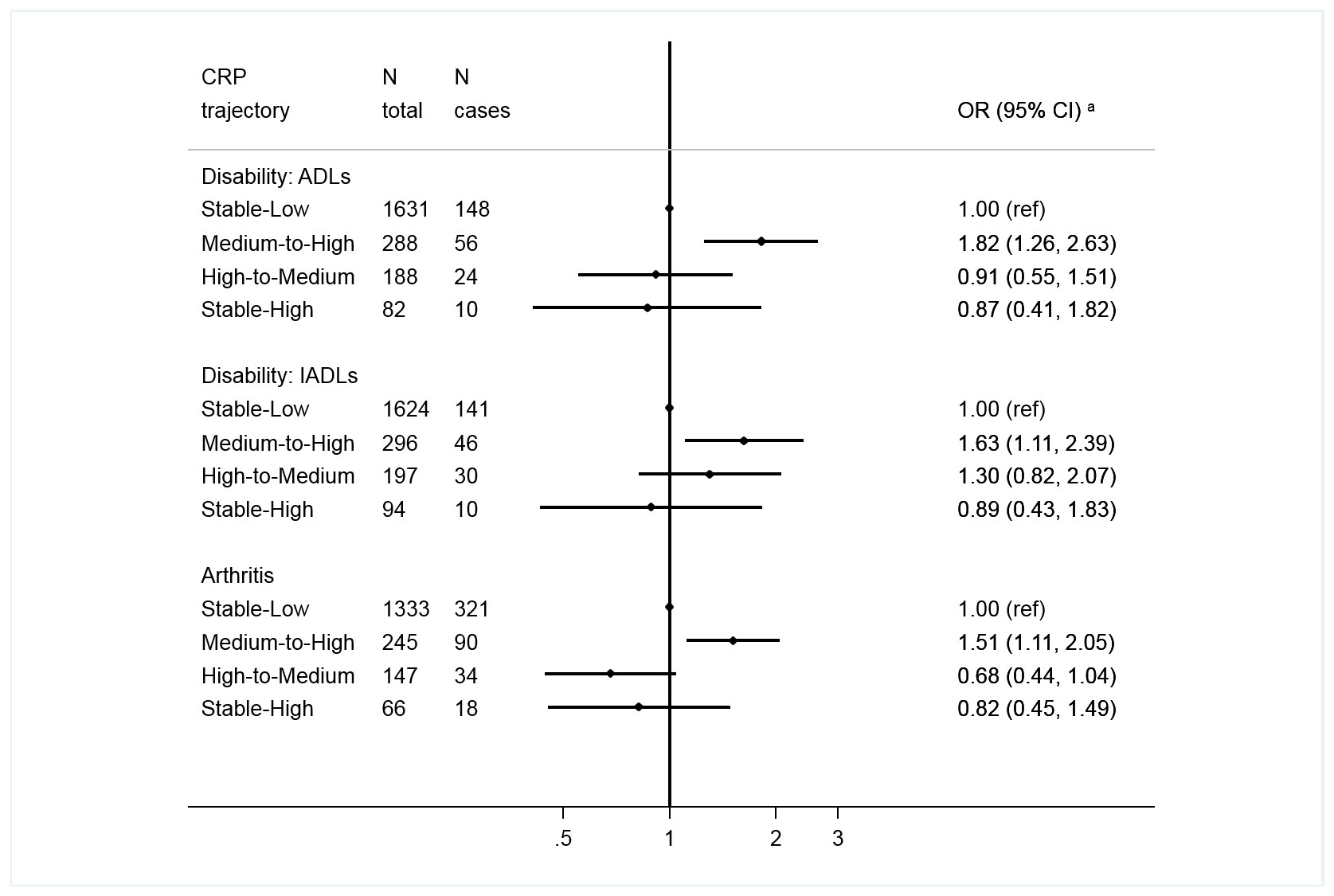


^a^ All ORs are adjusted for sex and baseline age, body mass index, smoking status, physical activity level, educational level and use of anti-inflammatory drugs.

**Supplemental Figure 3**. Odds ratios (95% CI) for the associations between CRP trajectories and markers of cardiometabolic health at wave 6 (2012-2013) after excluding participants with respective dysfunction at baseline, the English Longitudinal Study of Ageing


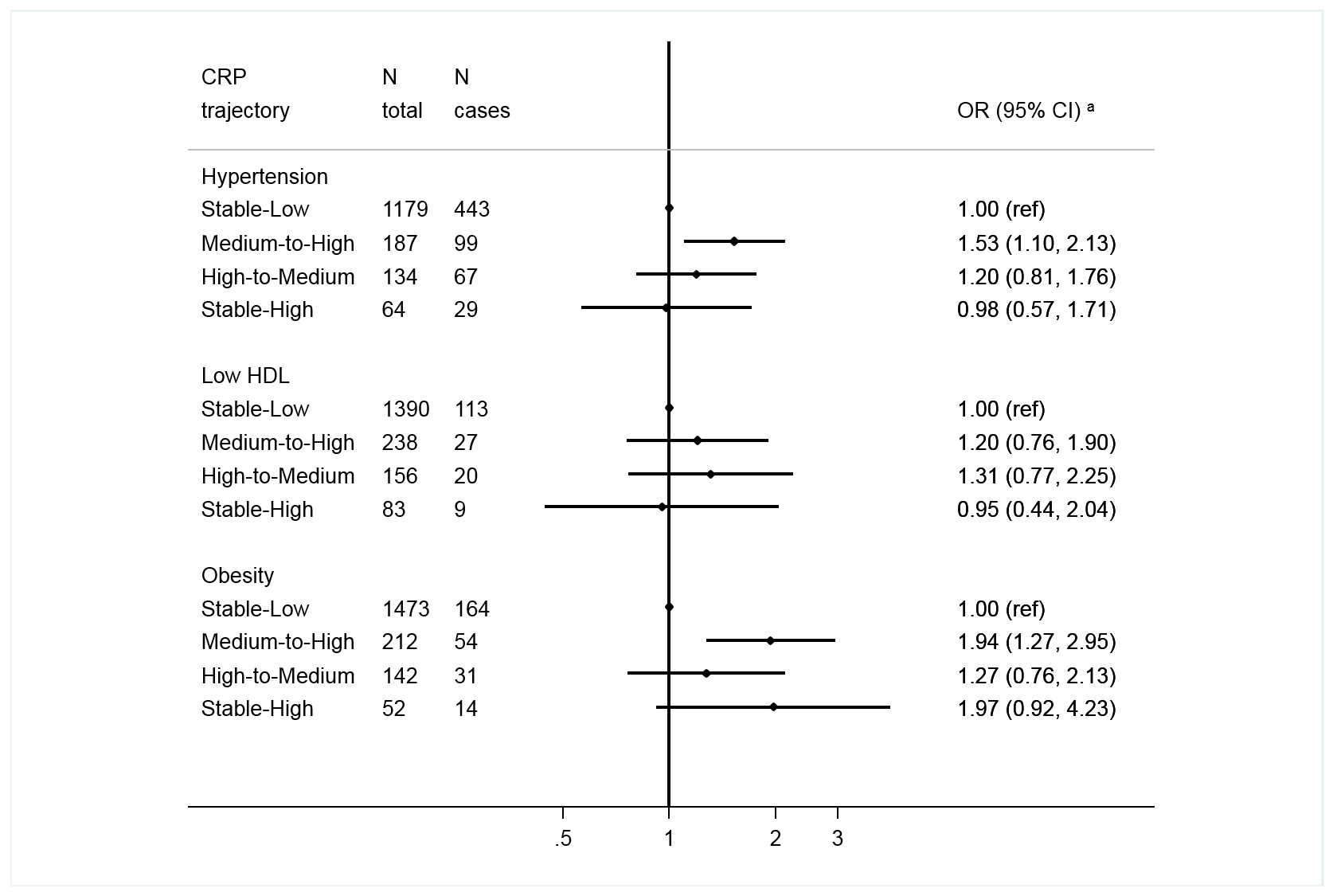


^a^ All ORs are adjusted for sex and baseline age, body mass index, smoking status, physical activity level, educational level and use of anti-inflammatory drugs.

**Supplemental Figure 4.** Odds ratios (95% CI) for the associations between CRP trajectories and mental health outcomes at wave 6 (2012-2013) after excluding participants with respective impairment at baseline, the English Longitudinal Study of Ageing


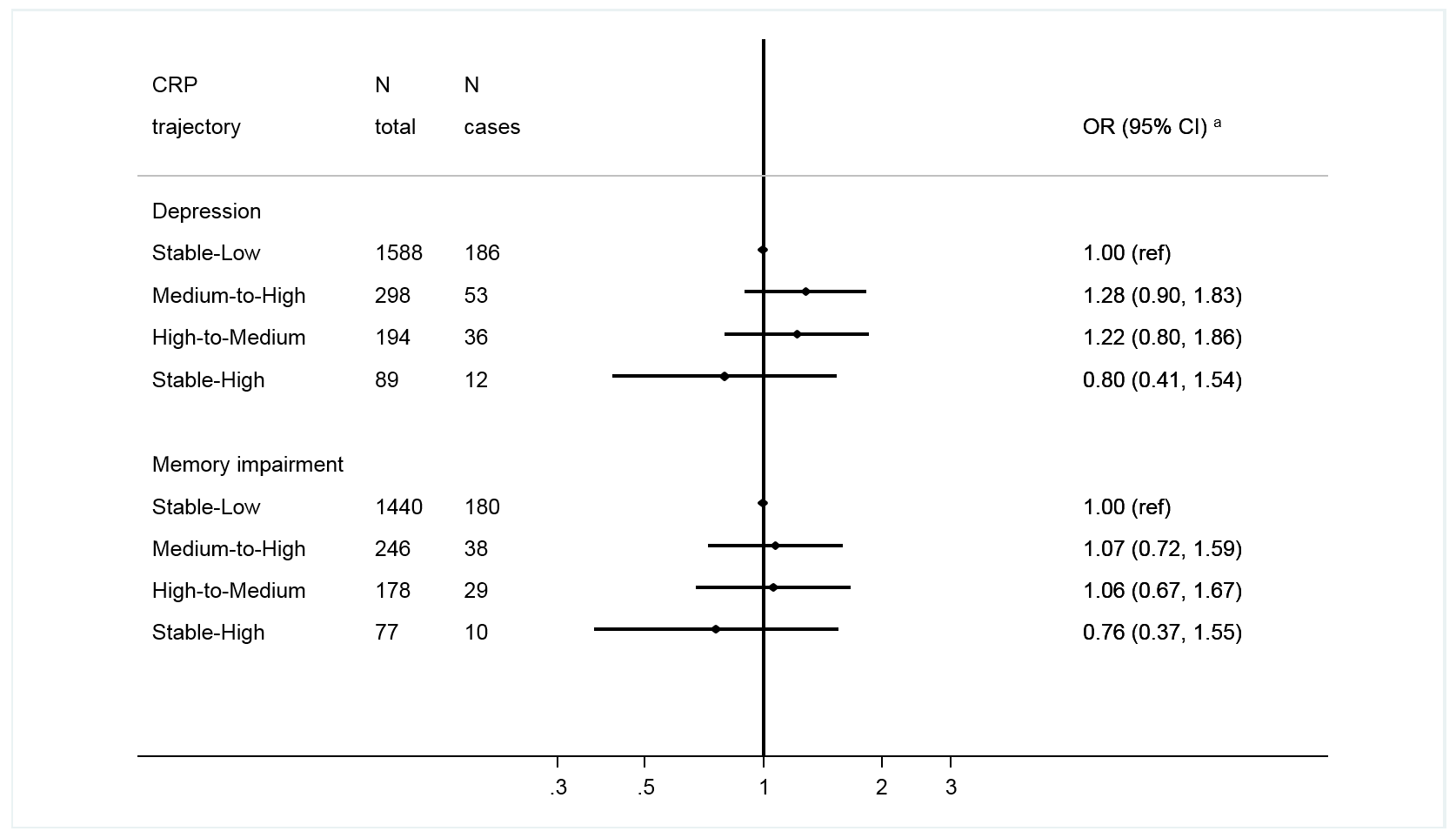


^a^ All ORs are adjusted for sex and baseline age, body mass index, smoking status, physical activity level, educational level and use of anti-inflammatory drugs.

**Supplemental Figure 5.** Relative risk estimates ^a^ (95% CI) for the associations between CRP trajectories and non-rare outcomes (>20%), the English Longitudinal Study of Ageing


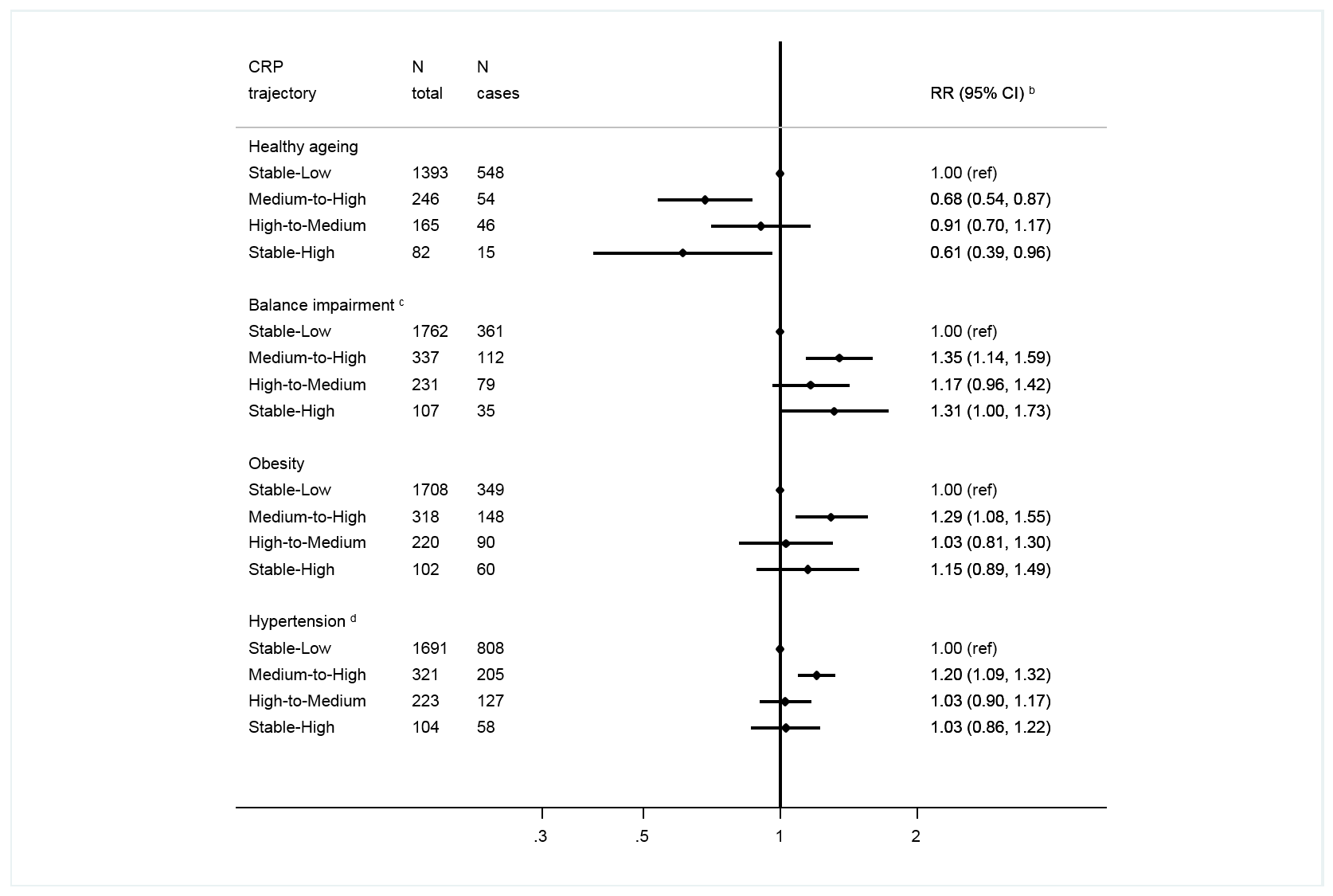


^a^ Relative risk estimates were obtained from Poisson regression with robust error variance

^b^ All RRs are adjusted for sex and baseline age, body mass index, smoking status, physical activity level, educational level and use of anti-inflammatory drugs.

^c^ RRs further adjusted for baseline arthritis

^d^ RRs further adjusted for baseline antihypertensive drug use
